# Quadricuspid pulmonic valve found on well exam

**DOI:** 10.1186/s40779-015-0037-2

**Published:** 2015-04-23

**Authors:** Stephen N Dunay, Robert A Roberge, Lena S Avedissian

**Affiliations:** Madigan Army Medical Center, 9040 Jackson Avenue, Tacoma, Washington 8431 USA

**Keywords:** Quadricuspid pulmonic valve, Radiology, Cardiology

## Abstract

Quadricuspid pulmonic valve (QPV) is almost always a benign anomaly and is therefore usually discovered incidentally on radiographic studies or post-mortem at autopsy. Because of its rarity, the true extent of the physiology of QPV is not fully understood, and the few reported cases of it may underestimate its physiological consequences. In this case, we report on a young active-duty solider who presented for a well check-up and was discovered on imaging to have a QPV. We also review the most recent literature and provide recommendations regarding the most effective diagnostic modalities.

**Electronic supplementary material:**

The online version of this article (doi:10.1186/s40779-015-0037-2) contains supplementary material, which is available to authorized users.

## Background

Quadricuspid pulmonic valve (QPV) is a rare congenital anomaly, occurring in approximately 1 in 400 to 1 in 2,000 people, with a 2:1 male to female ratio [1,2]. Unlike a bicuspid pulmonary valve, it rarely presents with clinical complications, such as valvular insufficiency or stenosis, and thus, it is most often a post-mortem finding [1,3,4]. Due to the common congenital origins of these two anomalies, a QPV is frequently associated with aortic valve anomalies; however, unlike a quadricuspid aortic valve, which often leads to stenosis or insufficiency, a four-leaflet pulmonary valve appears to have no negative consequences on pulmonary circulation [2-4]. To date, only two cases of QPV with stenosis have been reported, and the literature has described rare cases with evidence of pulmonary regurgitation and/or hypertension [4,5]. A few pathological studies have shown white patches of endocardial thickening associated with abnormal flow, attributed to an incompetent valve; however, this regurgitation is most often insufficient to cause dilation of the right ventricle or pulmonary artery [4].

Congenital cardiac anomalies associated with QPV have only been reported in 4% of cases [5]. They are also more prevalent than those associated with quadricuspid aortic valve, with an estimated ratio of 9:15. This malformation is also commonly associated with patent ductus arteriosus, atrial and ventricular septal defects, coarctation of the aorta, and pulmonary artery aneurysm [2]. Due to the anatomical location of the valve with respect to the thoracic wall, diagnosis by two-dimensional echocardiogram is difficult; however advances in cardiac imaging, including multidimensional computerized tomography (CT), transthoracic echocardiogram (TTE), and magnetic resonance imaging (MRI), have allowed for effective, non-invasive means of diagnosis [2,6]. Abnormal function has been reported in approximately 4% of cases, mostly in the form of insufficiency following stenosis [2]. Most commonly, in approximately 60% of patients, the anomaly presents as three normal-sized leaflets and one small leaflet, and the accessory cusp is frequently deformed, shrunken, or fenestrated [4,5]. 15% of patients present with two normal and two small leaflets, 12% with four leaflets of equal sizes, and the remainder with four different-sized leaflets [5].

## Case presentation

Our case involves a 20-year-old male active Army soldier who presented for a Special Forces qualification physical. He denied any symptoms, including palpitations, dyspnea, paroxysmal nocturnal dyspnea, and orthopnea. His only past medical history included an unspecified, benign murmur during his childhood. He admitted to smoking almost a pack of cigarettes a day but was otherwise a very healthy individual who exercised regularly, and he reported no family history of cardiac disease. His physical exam was unremarkable except for a grade III systolic murmur heard at the right upper sternal border, and EKG demonstrated a regular heart rate and rhythm, with right axis deviation and non-specific T-wave changes.

**Figure 1:**
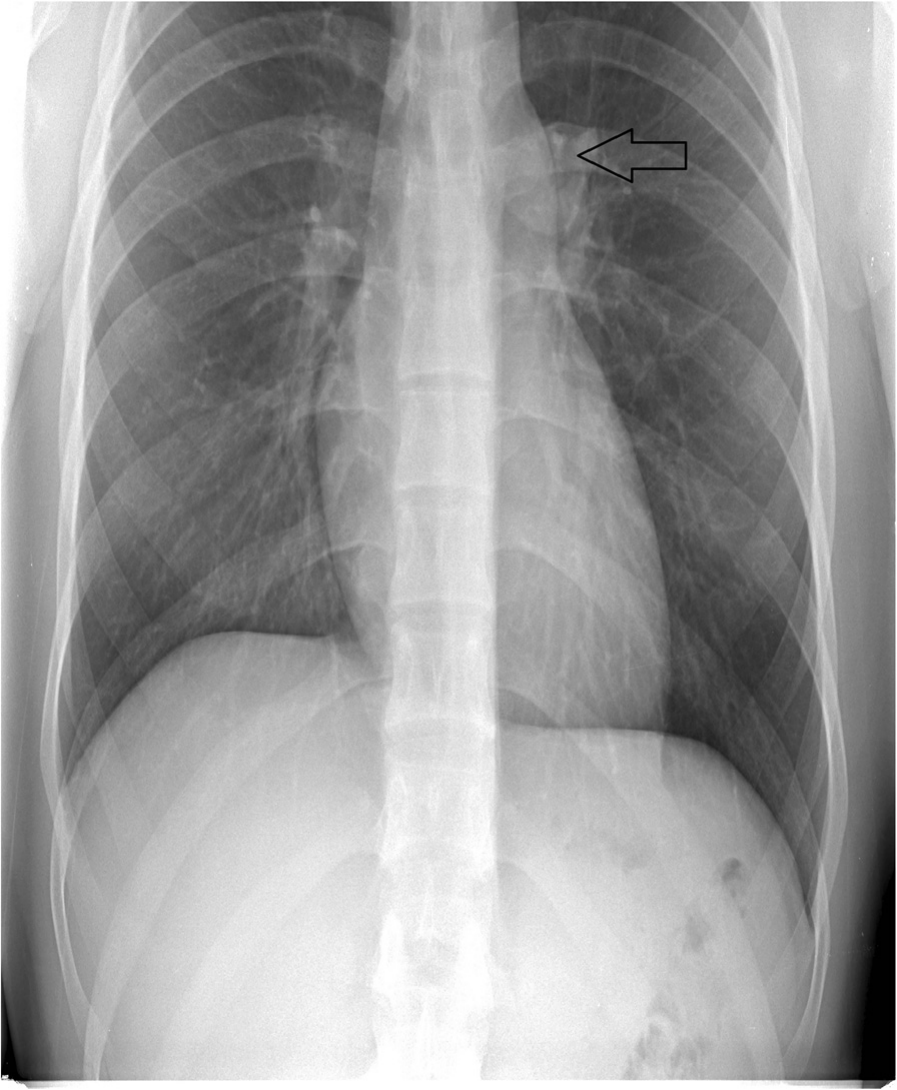
Chest X-ray, showing enlargement of the main pulmonary artery.

A routine chest X-ray, required for Special Forces eligibility, demonstrated no acute cardiopulmonary disease; however, an enlarged main pulmonary artery was observed, suggesting underlying pulmonary arterial hypertension or post-stenotic dilatation (Figure 1). Due to the abnormal finding on chest X-ray, the patient was sent for transthoracic echocardiogram, which demonstrated a normal left ventricular size with an ejection fraction of 60% - 65% and a mildly dilated right ventricle and mild right atrial enlargement, as well as moderate pulmonary regurgitation with a broad-based jet and an end-diastolic pulmonary artery pressure of 3 mmHg (Additional file 1).

**Figure 2:**
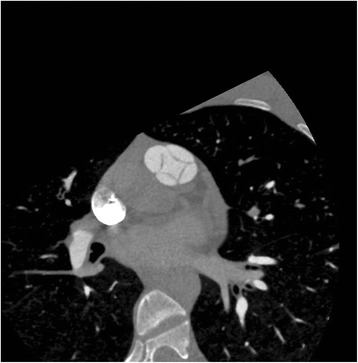
Low-dose prospectively gated multidetector computed tomography (MDCT) right ventriculogram, showing a QPV, a patent foramen ovale, and a normal right ventricular volume.

In preparation for cardiac CT with ventriculogram, the patient was administered 100 mg atenolol for heart rate control down to 46 beats per minute and 0.4 mg sublingual nitroglycerin for coronary vasodilation. The patient was placed in a 64-slice multidetector CT scanner and administered 70 ml Isovue-370 contrast intravenously. CT demonstrated a QPV with four equal-sized leaflets, borderline dilation of the right ventricle, a patent foramen ovale with the passage of a small amount of contrast across the atrial septum, and normal coronary arteries with left dominance (Figure 2).

Despite the patient’s asymptomatic presentation, he decided not to complete the Special Forces physical and did not submit a qualification packet. He was set up for a routine annual follow up with a cardiologist.

## Discussion

Embryologically, the semilunar valves are formed by mesenchymal outgrowth from proliferations of the two bulbar ridges and the intercalated valvular swellings [5]. Abnormal cusps may be formed by abnormal proliferation in the common trunk and aberrant fusion of the aortopulmonary septum [7].

The pulmonic valve, unlike the aortic valve, cannot be visualized in the short-axis view, making it difficult to determine the number of cusps of the semilunar valves by TTE [5]. However, TEE can be used to examine the pulmonic valve in the short-axis view, which is best appreciated with anteroflexion of the probe between 135° and 145° [5]. Due to the difficulty of visualization, retrospective EKG-gated CT angiography is the ideal diagnostic method because it permits the visualization of not only normal valve morphology but also congenital and acquired structural abnormalities, including direct observations of the number, thickness, and opening and closing of the leaflets and the presence of valve calcification [3].

Cardiac CT has been compared to MRI and has been shown to be more effective due to its better spatial resolution and the ability to acquire a multiphase data set of the heart in less than 15 seconds [3].

An important consideration in patients with QPV who also have aortic valve anomalies is the appropriateness of the Ross procedure, which involves the use of a pulmonary autograft for aortic valve replacement [3]. This procedure is particularly beneficial because it allows for the replacement of the diseased aortic valve without the need for systemic anticoagulation [3]. It has been proven useful in congenital aortic valve malformations when a tricuspid pulmonary valve of regular anatomy and function is used; however, because the hemodynamics of QPV are poorly understood, it is not an ideal candidate for autograft.

Although rare, the most common pathological finding associated with QPV is pulmonary regurgitation (PR) [5]. It is typically corrected through either pulmonary valve replacement or valve repair [8]. In one case, tricuspidization of the QPV has proven effective in correcting PR [8].

## Conclusion

Although it is currently considered a rare clinical finding, the prevalence of QPV is likely to increase due to advances in imaging technology. It has been proposed in the literature that a quadricuspid pulmonic valve cannot operate at maximum efficiency; however, in most cases, this finding has been clinically benign [4]. Multidetector cardiac CT appears to be the most effective diagnostic method due to its excellent spatial resolution [3]. Although QPV is considered to be clinically unimportant, it may be an underestimated contributor to pulmonary hypertension and heart failure due to its mostly post-mortem diagnosis.

## Consent

Written informed consent was obtained from the patient prior to the publication of this Case Report. A copy of the written consent is available for review by the Editor-in-Chief of this journal.

## Additional file

Additional file 1:
**Transthoracic echocardiogram demonstrating a mildly dilatedright ventricle and mild right atrial enlargement, as well as moderate pulmonary regurgitation.**
