# Erratum: Quadricuspid pulmonic valve found on well exam

**DOI:** 10.1186/s40779-015-0043-4

**Published:** 2015-06-27

**Authors:** Stephen N. Dunay, Eric A. Roberge, Lena S. Avedissian

**Affiliations:** Madigan Army Medical Center, 9040 Jackson Avenue, Tacoma, WA 8431 USA

## Erratum

After publication of this work [1], we noted the second author’s name was incorrect as Robert A Roberge. The author’s correct name should be Eric A Roberge.
